# A systematic review of the effectiveness of community-based interventions aimed at improving health literacy of parents/carers of children

**DOI:** 10.1177/17579139231180746

**Published:** 2023-06-29

**Authors:** SL Belfrage, M Husted, SDS Fraser, S Patel, JA Faulkner

**Affiliations:** University of Winchester, Winchester SO22 4NR, UK; University of Winchester, Winchester, UK; University of Southampton, Southampton, UK; Southampton Children’s Hospital, Southampton, UK; University of Winchester, Winchester, UK

**Keywords:** health literacy, systematic review, health improvement, meta-analysis

## Abstract

**Aim:**

The aim of this systematic review was to examine the effectiveness of community-based health literacy interventions in improving the health literacy of parents.

**Methods:**

A systematic review of six databases – MEDLINE, PsycINFO, CINAHL, Cochrane Library, Embase, and Education Source – was conducted to identify relevant articles. Risk of bias was assessed using version two of the Cochrane risk of bias tool for randomised controlled trials or the Cochrane collaboration risk of bias in non-randomised studies of interventions. The study findings were grouped and synthesised following the synthesis without meta-analysis framework.

**Results:**

Eleven community-based health literacy interventions for parents were identified. Study design included randomised controlled trials (*n* = 4), non-randomised studies with comparison group (*n* = 4), and non-randomised studies without a comparison group (*n* = 3). Interventions were delivered digitally, in person or a combination of the two. The risk of bias was high in over half the studies (*n* = 7). The main findings of the studies showed some potential for both in person and digital interventions to increase parental health literacy. Studies were heterogeneous preventing a meta-analysis.

**Conclusion:**

Community-based, health literacy interventions have been identified as potential methods for enhancing parental health literacy. Due to the small number of included studies and their potential for bias, these results must be interpreted with caution. This study emphasises the need for additional theory and evidence-based research on the long-term effects of community interventions.

## Introduction

Addressing health inequalities is a global health priority. When children of disadvantaged parents/caregivers are compared to their peers, health inequalities such as obesity and long-term health conditions are evident.^1^ Increased emergency hospital use is also associated with deprivation.^2^ Providing each child with the best possible start in life is crucial to lowering health disparities across the life course. Accordingly, in trying to achieve the best possible start in life for their child parents/caregivers must navigate a complex landscape of health-related information, healthcare provisions, and health systems.

Assessing a parent’s health literacy is one way to capture their ability to manage these resources.^3^ A widely accepted definition of health literacy is ‘the personal characteristics and social resources needed for individuals and communities to access, understand, appraise and use information and services to make decisions about health’.^4^ Children with long-term health conditions who have parents/caregivers with low health literacy have higher paediatric emergency department use and increased non-urgent visits than children with long-term conditions whose parents/caregivers have adequate or above health literacy.^5^ Improved health literacy has been shown to empower individuals and communities to navigate health services effectively and self-manage their health needs, therefore reducing the burden on health and social care services.^6^ A prior systematic review indicated parental health literacy is closely related to child health and recommended interventions to reduce the literacy demands on parents would be beneficial.^7^

Health literacy research has primarily focused on the individual; however, some researchers have expressed a view to widen its conceptualisation beyond the individual to the family, community, and society levels.^8^ Although community-based interventions such as peer support groups and educational workshops in informal settings can empower individuals to make positive changes to improve their own and their family’s health and wellbeing,^9^ they often focus on managing long-term conditions, making them less generalisable to general community (non-clinical) populations managing their own and their family’s day to day health and wellbeing or acute health problems.

Despite a move towards a more comprehensive approach to health literacy interventions with the proposed health literacy intervention model, there are a variety of intervention designs and no conclusive evidence supporting the individual components of an effective community-based health literacy intervention for groups, including parents.^10^ Several studies have developed community interventions such as short message service (SMS)-based mobile interventions, and co-produced, community lead support groups to improve parental health literacy, however the effectiveness of these interventions has not been systematically and comprehensively synthesised.^11–13^ Accordingly, the aim of the present study was to conduct a systematic review to examine the extent to which community-based health literacy interventions are effective at improving the health literacy of parents/caregivers. This systematic review will provide evidence to support the effective development of future parent-focused community-based health literacy interventions.

## Methods

This systematic review was guided by the Cochrane Handbook for Systematic Reviews of Interventions^14^ and reported according to the Preferred Reporting Items for Systematic Reviews and Meta-Analyses (PRISMA) checklist^15^ and the Synthesis Without Meta-Analysis (SWiM) guidelines.^16^ The review protocol was registered with the International Prospective Register of Systematic Reviews (PROSPERO), registration number: CRD42021241503.

### Eligibility criteria

Study eligibility criteria were defined a priori. The PICOS (population, intervention, comparison, outcomes, setting) framework was used.^15^ Supplemental Table 1 provides a summary of the inclusion and exclusion criteria. Full details of the inclusion and exclusion criteria are included in the review protocol. All quantitative and mixed method study designs were considered, including experimental, quasi-experimental, and non-experimental designs; however, only quantitative data was synthesised. All included studies had a quantitative component with a validated health literacy measure.

### Search strategy

One researcher (S.L.B.) independently searched MEDLINE (EBSCO), PsycINFO (EBSCO), CINAHL (EBSCO), Cochrane Library, Embase and Education Source (EBSCOhost). No date restrictions were applied. Basic and medical subject headings (MeSH) searches were performed within each database. Search terms included ‘health literacy’, ‘community’, ‘parents’, and related terms (see Supplemental Appendix A). Grey literature searching of the National Institute for Health and Care Excellence (NICE), King’s Fund, Public Health England, British Library, Health Literacy UK, and Open Grey websites were conducted, using the search terms: ‘health literacy’ and ‘community’. Studies that met the inclusion criteria and published in the English language were considered for inclusion. Searches were initially conducted in March/April 2021 and database searches were re-run in September 2021 prior to the final analysis to ensure no new publications had arisen in the intervening 6 months.

### Study selection

One researcher (S.L.B.) exported all publications from the databases that met the search criteria into Covidence. Two reviewers (S.L.B. and J.A.F.) independently screened the titles and abstracts and recorded them as included/excluded/unsure. Reviewers then discussed any conflicts in screening decisions and either included or excluded said paper. A third reviewer (M.H.) carried out integrity checks with regard to the application of inclusion/exclusion criteria.

### Data extraction and synthesis

A single author (S.L.B.) extracted the following data from each included study using a predefined data extraction table: title, author(s) (year), country, study aim (research question), study design, sample and setting, contents of intervention, outcomes (including health literacy measure), and limitations. The *I*^2^ test was used to test for heterogeneity between studies.

This review used the SWiM framework to synthesise results from included papers. SWiM comprises a nine-item framework facilitating structured synthesis for systematic reviews where heterogeneity of papers prevents undertaking a meta-analysis.

The studies were grouped according to study design (randomised controlled trial (RCT), non-randomised with comparison group, and non-randomised without comparison group). Pooling by health outcome was not feasible due to the number of distinct health conditions included across the studies, nor was pooling by intervention due the range in complexity, intensity, and length of interventions. The standardised mean difference (SMD) was the standardised metric used to allow for synthesis. SMD was used as a measure of effect size (*d* = 0.2, 0.5, and 0.8 for small, medium, and large effects, respectively)^17^ and was calculated for studies with a control group providing the necessary pretest and post-test data. Following the SWiM reporting items, a forest plot was created to show the individual effect size of interventions, no combined measure of effect was reported due to heterogeneity.

### Risk of bias assessment

For RCTs, the Cochrane risk of bias tool for randomised trials (ROB-2)^18^ was used. Each domain encompasses three to seven signalling questions, each of which is assigned a risk of bias of ‘low’, ‘high’, or ‘some concerns’. For non-randomised studies, the risk of bias in non-randomised studies – of interventions (ROBINS-I)^19^ – was used. This tool assesses bias relating to confounding, selection, classification of interventions, deviations from intended interventions, missing data, measurement outcomes, and reporting. Overall judgement for the ROBINS-I assessment is ‘low’, ‘moderate’, ‘serious’, ‘critical’, or ‘no information’. Two reviewers (S.L.B. and J.A.F.) completed the risk of bias assessment for all included studies independently, and discrepancies were resolved through discussion. Where consensus was not clear, a third reviewer (M.H.) oversaw discussions. Both the proposed domain-level and overall risk-of-bias judgements were produced by the risk of bias tool algorithms.

## Results

### Study selection

Electronic database searching identified 10,091 papers (Supplemental Figure 1). One additional unpublished study was identified from grey literature; however, despite contacting the author the full article was unobtainable. There were 5581 unique publications after removing duplicates of which 5439 did not meet inclusion criteria on title and abstract screening. Of the 142 papers retrieved for full text review, 131 did not meet the inclusion criteria. Eleven papers were included in the final review (Supplemental Table 2).

### Study characteristics

Supplemental Table 2 summarises the included studies. Four studies were RCTs, and seven were non-randomised. All were published between 2017 and 2021.^11–13,20–27^ Studies were conducted in a range of countries: three in middle income,^20–22^ eight in high income.^11–13,23–27^ Interventions were conducted in two main settings: community,^13,26^ including sports clubs,^24^ university campus,^25^ and outpatient;^12,22^ and digital^11,20,21,23^ or a combination of these.^27^ Tools and techniques used to develop health literacy skills included group discussions, text messages, signposting to additional resources, and handouts.

There was considerable heterogeneity between included RCTs (*I*^2^ = 80%) and between included non-randomised studies with comparison groups (*I*^2^ = 98%).

### RCTs

The four RCTs (total participants *n* = 566) focused on oral health,^20^ epilepsy,^21^ mental health,^11^ and childhood immunisation.^12^ The one in person intervention was conducted by the researcher.^12^ In one study, the gender of the participants was not indicated,^20^ while in another, only females were recruited,^12^ and in two studies, both male and female participants were recruited.^11,21^ Furthermore, study inclusion criteria included pregnant women aged 18 years or older, and gestational weeks 29–33;^12^ youth with epilepsy;^21^ low socioeconomic level^20^ and age of the child.^11^

### Non-randomised studies with comparison group

The four included intervention studies (total participants *n* = 875) focused on mental health,^24,25^ nutrition,^23^ and physical activity.^22^ In-person interventions were conducted by the researcher.^22,24,25^ In one study, the gender of participants was not specified;^23^ in another, only females were recruited;^22^ and in two investigations, a higher proportion of female participants than male participants was observed.^24,25^ All participants were recruited from community settings. One study was conducted in a middle-income country^22^ and three in high-income countries.^23–25^

### Non-randomised studies without comparison group

The three included intervention studies (total participants *n* = 150) focused on traumatic brain injury,^27^ maternal health,^28^ and oral health.^26^ The length of the intervention was unclear in two studies.^13,27^ The intervention ‘mumspace’ was delivered for 2 h each week for 6 months and ‘parent university’ was delivered for 2 h each week for 12 weeks.^13^ The oral health intervention was delivered as a single 1 h presentation.^26^ Face-to-face interventions were carried out by a paediatric dentist or registered nurse,^26^ a group of parents and health professionals,^13^ and a researcher at the outset, then by patient navigators who were certified medical professionals.^27^ All studies were conducted in high-income countries,^13,26,27^ and included the recruitment of only females,^13^ or a combination of both females and males.^26,27^

### Risk of bias

All RCTs had an overall ‘high’ risk of bias (Supplemental Figure 2), predominantly arising from concerns about outcome measurement (in three studies)^11,12,20^ and deviations from intended interventions in one study.^21^ This was due to the assessment of the outcome potentially being influenced by knowledge of the intervention and the assessment outcome potentially being influenced by knowledge of the intervention received. For non-randomised studies with a comparison group, the overall risk of bias was ‘high’ for two studies,^23,24^ ‘moderate’ for one study,^25^ and ‘low’ for one study (Supplemental Figure 3).^27^ For non-randomised studies without comparison group the overall risk of bias judgement was ‘high’ for one study,^26^ ‘moderate’ for one study,^13^ and ‘low’ for one study (Supplemental Figure 4).^27^

### SWiM

The forest plot in Supplemental Figure 5 highlights greater benefit from community-based health literacy interventions relative to comparison groups for all eligible RCTs (SMD = 0.53; median effect size = 0.32) and non-randomised studies (SMD = 1.03; median effect size = 0.67) studies. The effect size distribution is positively skewed in both study designs with the mean being larger than the median. The outlier showed a significantly larger effect size when compared to other included studies.^25^ With the outlier removed, the mean effect size for non-randomised studies = 0.48 and the median effect size for non-randomised studies = 0.65. Using the Cohen’s^29^ *d* effect size interpretation, the RCTs show a medium positive effect (*d* = 0.53) and the non-randomised studies show a large positive effect (*d* = 1.03), with the outlier being removed the non-randomised studies show a small positive effect (*d* = 0.48).

## Health Literacy Outcomes

### RCTs

The four RCT interventions showed a significant increase in health literacy.^11,12,20,21^ All studies met their reported sample size requirement; however, health literacy was only a primary outcome of one study.^11^ The self-reported eHealth Literacy Scale (eHEALS)^30^ was used in two studies to measure outcomes.^20,21^ One study^11^ used the validated subjective Mental Health Literacy Scale,^31^ and one study^12^ reported findings from the adapted Scale of Health literacy.^32^ In three of these studies, statistically significant improvements of 9.87%, (intervention delivered via text message),^20^ 12.1% (intervention delivered in person),^12^ and 42% (web-based intervention),^21^ were reported for the intervention group compared to control. One study reported increased mental health literacy in the intervention group compared with the control as an increased estimated mean difference, 0.99 points; 95% confidence interval (CI).^11^ Furthermore, details of the interventions and results are provided in Supplemental Table 2.

### Non-randomised studies with comparison group

The four non-randomised studies reported statistically significant increases in health literacy.^22–25^ All four studies stated health literacy as a primary outcome. One study^22^ included two measures of health literacy (both objective), via the Short Test of Functional Health Literacy in Adults (S-TOFHLA)^33^ and the Rapid Estimate of Adult Literacy in Medicine (REALM).^34^ Parents in the intervention group of one study^22^ had a baseline mean S-TOFHLA score of 26.2 and a statistically significant increase to 29.18 8 weeks after the intervention (*p* < .001). A second tool was utilised in the study;^22^ the REALM which reported a baseline mean (standard deviation (SD)) of 60.88 (2.78) which following the intervention increased to 62.18 (2.48). One study^23^ reported improvements of nutrition literacy in the intervention group of 6.1%, by the adapted Nutrition Literacy Assessment Instrument.^35^

Two studies reported significant improvements in mental health literacy utilising an adapted version of the mental health literacy scale,^31^ 9.7% and 7.1% for the intervention group compared to the comparison group.^24,25^

### Non-randomised studies without comparison group

A significant increase to health literacy was only made in the low health literacy subgroup in one study^13^ using the Newest Vital Sign measure.^36^ In one study,^26^ significant increase in correct answers from pretest (68.8%) to post-test (92.6%) on the following topics were reported using the Upper Peninsula Oral Health Literacy Assessment Survey: ‘when to take a child to the dentist for the first time’, ‘germs can cause cavities’, ‘smoking in the home increases cavities’, and ‘care givers can pass germs to children’. Gains in health literacy measured using the short-form Health Literacy Scale were not statistically significant at 3-, 6-, or 12-month follow-ups following the telephone-based intervention.^27^

## Discussion

The purpose to this review was to examine how effective community-based health literacy interventions are at increasing the health literacy of parents. This systematic review identified a total of 11 studies of interventions delivered in a variety of settings, by several methods and focusing on a range of health topics. Although no definitive conclusion of the effectiveness of community-based interventions can be drawn, there are suggestions of improvement in many of the studies included in this review. However, the review has highlighted concerns with the methodological quality due to the high risk of bias of several included studies and has brought into question whether the health literacy measurement tools used met the needs of assessing the interventions outcomes. Also acknowledged is the need for additional theory and evidence-based research on the long-term effects of community-based interventions.

Depending on the study design, two distinct tools were used to assess the risk of bias. While RCTs are considered the gold standard for effectiveness research, blinding is challenging in intervention studies, contributing to their overall higher risk of bias compared to non-randomised studies in this review. Although there is no definitive conclusion of the overall effectiveness of community-based interventions, there are suggestions of improvement in parental health literacy in many of the studies included in this review.

To the research team’s knowledge, this is the first systematic review to explore the effectiveness of community-based health literacy interventions aimed specially at parents. Of the eleven studies included in this review, eight different health topics were included, and eight unique tools were used to capture change in health literacy. These variations in outcome measures and breadth of the topics included in the interventions including, mental health, oral health, and nutritional health worsen issues around the ability to synthesise findings and provide definitive conclusions. Alongside the high number of different health literacy measurement tools used, only two of the included studies described the theoretical underpinning of the intervention. Research suggests that interventions grounded in theory result in improved outcomes, because theories provide a systematic process to understand a phenomenon by explaining why, and under what circumstances, certain outcomes occur.^37^ An alternative review, looking at interventions aiming to improve health literacy and health behaviour, drew similar conclusions.^38^ The conclusion therefore from this review, and similar reviews, is that adopting a coherent link between health literacy and behaviour change theories and frameworks within research will result in higher quality and more effective interventions.

This review highlights the need for researchers to use appropriate health literacy measurement tools which meet the needs of their intervention study. The requirement for appropriate health literacy measurements tools is illustrated by the study using the self-reported eHEALS tool to measure parents’ ability to find critique and use electronic health information.^20^ The study found a significant increase at 6 months follow-up in the intervention group even though the aim of the messages sent during the intervention did not include critiquing health information. This illustrates the potential risk of response bias present when using self-reported tools. Response bias can occur due to social desirability, with females being more likely to respond in a socially desirable manner, and where data on gender was available, it indicated more female than male participants.^39^ While self-reporting subjective instruments are easier to administer, they often lack a strong theoretical grounding. For example, some participants might not have an accurate insight into their own abilities. Evidence suggests that objective and subjective health literacies were weakly associated with each other.^40^ This research showed that individuals with high subjective health literacy scores did not necessarily have the practical skills to assess and critique health information, which would have been captured by an objective health literacy measure.^40^

Another issue regarding the ability of the health literacy measurement being appropriate to the intervention design was the use of the REALM, S-TOFHLA, and the Newest Vital Sign (NVS-UK), all of which are screening tools designed to be used to assess levels of health literacy and not as a tool to measure change. For instance, REALM’s restricted focus on health-related word pronunciation prevents it from being an effective tool for measuring changes in health literacy.^41^ Furthermore, the ‘ceiling effect’ is an additional measurement limitation that occurs when the highest possible or near highest score on a test is achieved, thereby reducing the likelihood that the test instrument has accurately measured the intended domain.^42^ Specifically, this was seen in studies where mental health literacy at baseline was high, meaning there was limited opportunity to increase this score with the intervention; therefore, the effectiveness of the intervention needs to be interpreted with caution.^24,25^

The findings from this review have implications for practice as well as research. For commissioners of health and social care services, it is noteworthy that the digital delivery of health literacy interventions provided a lower cost and a less resource intensive alternative to face-to-face educational interventions. The case was made for a universal text-messaging intervention to increasing parental mental health literacy as a way of reducing stigma related to accessing mental health support and taking a preventive approach.^11^ In addition, for community-based organisations, it is worth highlighting that although there is no evidence of long-term impact, several studies showed improvement in health literacy after a brief one-time intervention.^24,25^ This suggests that one-time education-based health literacy sessions such as exploring where to access health information and discussing decision making processes could be beneficial to parents.

Although the results of many of the included studies were encouraging, in some cases data were only collected immediately before and after the intervention with no subsequent follow-up. The potential long-term impacts of these interventions cannot be assessed as a result. Evidence suggests that health literacy interventions can improve health literacy and lead to changes in health behaviours.^38^ However, just three of the included studies measured a behaviour outcome following the intervention. This highlights the need to invest in research into the long-term effects of health literacy interventions to measure both health literacy and the follow-on behavioural outcomes. Alongside the need of longitudinal study designs, studies that use health literacy as a primary outcome are also needed as this will allow for appropriate sample sizes being recruited/calculated thus meaning sufficient focus is made to health literacy as a primary focus, and more robust conclusions can be drawn from the findings.

## Strengths and Limitations

It is important to contextualise the review considering the study strengths and limitations. One of the strengths of this systematic review was the use of a broad search strategy which allowed for inclusion of all community-based studies with a measure of health literacy without a limitation of study design, including a search of the grey literature. The systematic review was registered on PROSPERO allowing for transparency in the review process. SWiM guidelines were followed to promote clarity in the methods used in this review.^16^ Two reviewers were used during the study selection process which has shown to increase the number of included studies in systematic reviews. The decision to include a limit on the language of included studies (English) might have resulted the omission of relevant studies produced in other languages, which could have provided different results. There is potential for publication bias, in which research with favourable results are more likely to be published and published faster than those with negative results.^43^ Furthermore, because of the high levels of heterogeneity across the studies (including the measures and outcomes), a meta-analysis was not possible. Although the SWiM guidelines were followed, the study synthesis should be treated with caution due to the limited number of included studies and lack of quality assessment which limits the external validity of the findings.^16^

## Conclusion

Although this review does not make a definitive conclusion on the effectiveness of community-based health literacy interventions for parents, the findings suggest that community-based health literacy interventions delivered in person or digitally may improve parental health literacy outcomes. However, the risk of bias in over half of the included studies, the range of health literacy domains covered, the use of different measurement tools, and the heterogeneity means caution is needed when interpreting the results.

More theoretically informed community-based health literacy interventions for parent/caregivers are required as theories present a systematic approach to understanding how and why an intervention facilitates change. A move towards applying validated objective performance-based health literacy measurement tools would strengthen the evidence base of health literacy outcome measures and potentially allow for meta-analysis. Achieving this alongside longer follow-up for intervention studies would provide essential knowledge on the potential long-term impact and behaviour change outcomes of community-based health literacy interventions. Therefore, we call for studies with larger sample sizes and better data quality to improve the quality of evidence proving a stronger foundation to facilitate moving evidence into practice.

## Supplemental Material

sj-docx-1-rsh-10.1177_17579139231180746 – Supplemental material for A systematic review of the effectiveness of community-based interventions aimed at improving health literacy of parents/carers of children Supplemental material, sj-docx-1-rsh-10.1177_17579139231180746 for A systematic review of the effectiveness of community-based interventions aimed at improving health literacy of parents/carers of children by SL Belfrage, M Husted, SDS Fraser, S Patel and JA Faulkner in Perspectives in Public Health

sj-docx-2-rsh-10.1177_17579139231180746 – Supplemental material for A systematic review of the effectiveness of community-based interventions aimed at improving health literacy of parents/carers of children Supplemental material, sj-docx-2-rsh-10.1177_17579139231180746 for A systematic review of the effectiveness of community-based interventions aimed at improving health literacy of parents/carers of children by SL Belfrage, M Husted, SDS Fraser, S Patel and JA Faulkner in Perspectives in Public Health

sj-docx-3-rsh-10.1177_17579139231180746 – Supplemental material for A systematic review of the effectiveness of community-based interventions aimed at improving health literacy of parents/carers of children Supplemental material, sj-docx-3-rsh-10.1177_17579139231180746 for A systematic review of the effectiveness of community-based interventions aimed at improving health literacy of parents/carers of children by SL Belfrage, M Husted, SDS Fraser, S Patel and JA Faulkner in Perspectives in Public Health

sj-docx-4-rsh-10.1177_17579139231180746 – Supplemental material for A systematic review of the effectiveness of community-based interventions aimed at improving health literacy of parents/carers of children Supplemental material, sj-docx-4-rsh-10.1177_17579139231180746 for A systematic review of the effectiveness of community-based interventions aimed at improving health literacy of parents/carers of children by SL Belfrage, M Husted, SDS Fraser, S Patel and JA Faulkner in Perspectives in Public Health

sj-docx-5-rsh-10.1177_17579139231180746 – Supplemental material for A systematic review of the effectiveness of community-based interventions aimed at improving health literacy of parents/carers of children Supplemental material, sj-docx-5-rsh-10.1177_17579139231180746 for A systematic review of the effectiveness of community-based interventions aimed at improving health literacy of parents/carers of children by SL Belfrage, M Husted, SDS Fraser, S Patel and JA Faulkner in Perspectives in Public Health

sj-docx-6-rsh-10.1177_17579139231180746 – Supplemental material for A systematic review of the effectiveness of community-based interventions aimed at improving health literacy of parents/carers of children Supplemental material, sj-docx-6-rsh-10.1177_17579139231180746 for A systematic review of the effectiveness of community-based interventions aimed at improving health literacy of parents/carers of children by SL Belfrage, M Husted, SDS Fraser, S Patel and JA Faulkner in Perspectives in Public Health

sj-docx-7-rsh-10.1177_17579139231180746 – Supplemental material for A systematic review of the effectiveness of community-based interventions aimed at improving health literacy of parents/carers of children Supplemental material, sj-docx-7-rsh-10.1177_17579139231180746 for A systematic review of the effectiveness of community-based interventions aimed at improving health literacy of parents/carers of children by SL Belfrage, M Husted, SDS Fraser, S Patel and JA Faulkner in Perspectives in Public Health

sj-docx-8-rsh-10.1177_17579139231180746 – Supplemental material for A systematic review of the effectiveness of community-based interventions aimed at improving health literacy of parents/carers of children Supplemental material, sj-docx-8-rsh-10.1177_17579139231180746 for A systematic review of the effectiveness of community-based interventions aimed at improving health literacy of parents/carers of children by SL Belfrage, M Husted, SDS Fraser, S Patel and JA Faulkner in Perspectives in Public Health
