# Supplementary material for: A systematic review of the effectiveness of community-based interventions aimed at improving health literacy of parents/carers of children
Source: Perspect Public Health. 2023 Jun 29;145(1):25–31. doi: 10.1177/17579139231180746 (PMC11800687; doi:10.1177/17579139231180746)
Supplement: sj-docx-1-rsh-10.1177_17579139231180746 – Supplemental material for A systematic review of the effectiveness of community-based interventions aimed at improving health literacy of parents/carers of children [file sj-docx-1-rsh-10.1177_17579139231180746.docx]

**Supplemental Appendix A**

Example Search terms

MEDLINE-full text search strategy

(“health literacy” [MeSH] OR “health litera*”  [tiab] OR “rapid estimate of adult literacy in medicine and dentistry”[tiab] OR “berlin numeracy test” [tiab] OR “brief estimate of health knowledge and action” [tiab] Or “claim evaluation tools database” [tiab] OR “comprehension of 50 medical terms” [tiab] OR “comprehension health activities scale” [tiab] OR “critical health competence test” [tiab] OR “critical nutrition literacy instrument” [tiab] OR “diabetes numeracy test” [tiab]  OR “digital healthy diet test” [tiab] OR “eHealth literacy scale” [tiab] OR “evaluation tool development for food literacy programs” [tiab]  OR “food and nutrition literacy” [tiab] OR “general health numeracy test” OR “graph literacy scale” [tiab] OR “HIV literacy test” [tiab] OR “medical achievement reading test” [tiab] OR “medical data interpretation test” [tiab] OR “medical term recognition test”  [tiab] OR “medication literacy assessment” [tiab] OR “mental health-promoting knowledge” [tiab]  OR “numeracy understanding in medical instrument” [tiab] OR “parenting plus skills index” [tiab] OR “nutritional literacy scale” [tiab] OR “nutrition literacy assessment” [tiab]  OR “rapid estimate of adult literacy” [tiab] OR “subjective numeracy scale” [tiab] OR “single item screener” [tiab] OR “smoking media literacy” [tiab]  OR “test for ability to interpret medical information” [tiab] OR “the self perceived food literacy scale” [tiab] OR “water environmental literacy level scale” [tiab] OR “weight-specific health literacy instrument” [tiab]  OR “cancer health literacy test” [tiab] OR “cervical & breast cancer literacy assessment” [tiab]  OR “breast cancer literacy assessment tool” [tiab] OR “cervical cancer literacy assessment tool” [tiab]) AND (Parents [MeSH] OR Family [MeSH] OR Parent* [tiab] OR Mother* [tiab] OR Father* [tiab] OR famil* [tiab] OR Caregiver* [tiab] OR Guardian* [tiab] OR Maternal [tiab] OR Paternal [tiab])

Cochrane Library search strategy

(“health literacy”  [tiab] OR “rapid estimate of adult literacy in medicine and dentistry”[tiab] OR “berlin numeracy test” [tiab] OR “brief estimate of health knowledge and action” [tiab] Or “claim evaluation tools database” [tiab] OR “comprehension of 50 medical terms” [tiab] OR “comprehension health activities scale” [tiab] OR “critical health competence test” [tiab] OR “critical nutrition literacy instrument” [tiab] OR “diabetes numeracy test” [tiab]  OR “digital healthy diet test” [tiab] OR “eHealth literacy scale” [tiab] OR “evaluation tool development for food literacy programs” [tiab]  OR “food and nutrition literacy” [tiab] OR “general health numeracy test” OR “graph literacy scale” [tiab] OR “HIV literacy test” [tiab] OR “medical achievement reading test” [tiab] OR “medical data interpretation test” [tiab] OR “medical term recognition test”  [tiab] OR “medication literacy assessment” [tiab] OR “mental health-promoting knowledge” [tiab]  OR “numeracy understanding in medical instrument” [tiab] OR “parenting plus skills index” [tiab] OR “nutritional literacy scale” [tiab] OR “nutrition literacy assessment” [tiab]  OR “rapid estimate of adult literacy” [tiab] OR “subjective numeracy scale” [tiab] OR “single item screener” [tiab] OR “smoking media literacy” [tiab]  OR “test for ability to interpret medical information” [tiab] OR “the self perceived food literacy scale” [tiab] OR “water environmental literacy level scale” [tiab] OR “weight-specific health literacy instrument” [tiab]  OR “cancer health literacy test” [tiab] OR “cervical & breast cancer literacy assessment” [tiab]  OR “breast cancer literacy assessment tool” [tiab] OR “cervical cancer literacy assessment tool” [tiab]) AND (Parenting [MeSH] OR Family [MeSH] OR Parent* [tiab] OR Mother* [tiab] OR Father* [tiab] OR famil* [tiab] OR Caregiver* [tiab] OR Guardian* [tiab] OR Maternal [tiab] OR Paternal [tiab])
