# Supplementary material for: A systematic review of the effectiveness of community-based interventions aimed at improving health literacy of parents/carers of children
Source: Perspect Public Health. 2023 Jun 29;145(1):25–31. doi: 10.1177/17579139231180746 (PMC11800687; doi:10.1177/17579139231180746)
Supplement: sj-docx-4-rsh-10.1177_17579139231180746 – Supplemental material for A systematic review of the effectiveness of community-based interventions aimed at improving health literacy of parents/carers of children [file sj-docx-4-rsh-10.1177_17579139231180746.docx]

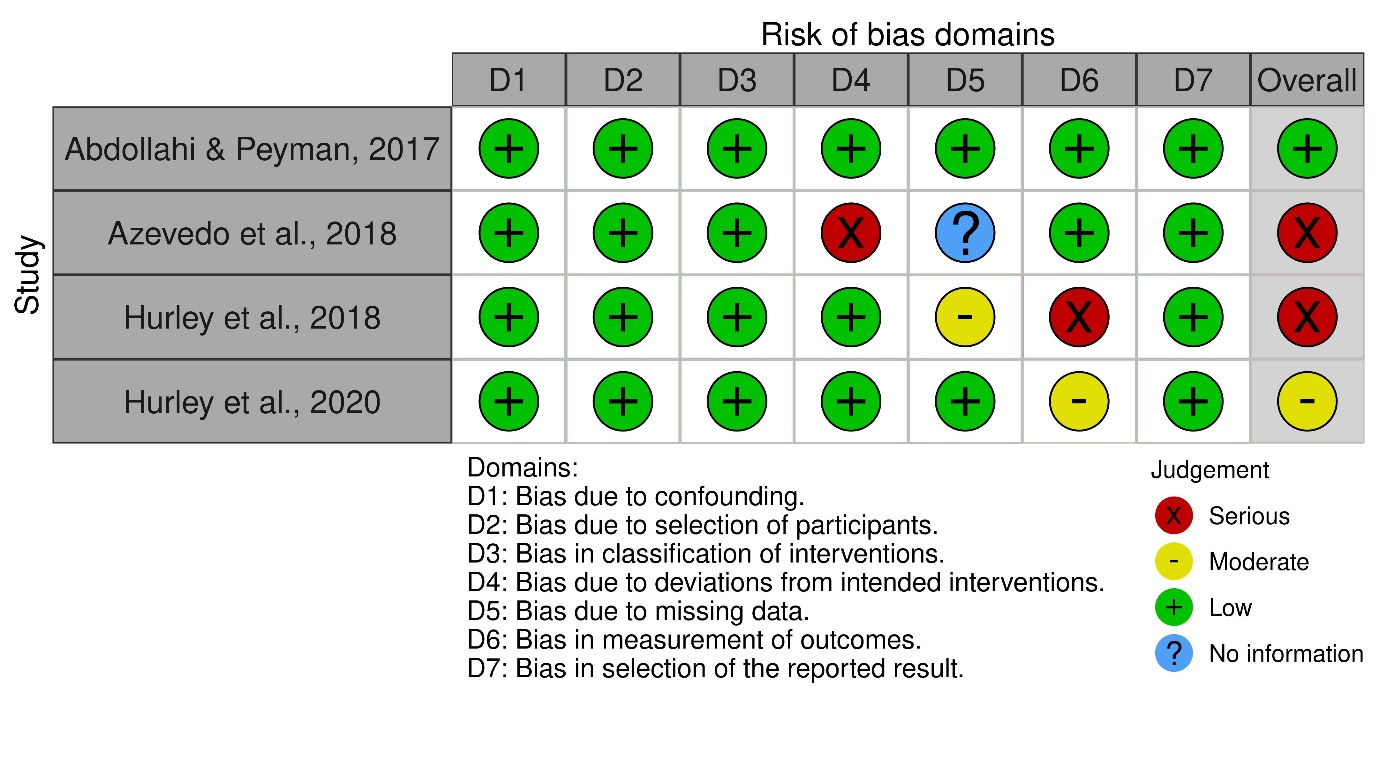


**Supplemental Figure 3.** Risk of bias plots for included non-randomised studies with a comparison group
