# Supplementary material for: A systematic review of the effectiveness of community-based interventions aimed at improving health literacy of parents/carers of children
Source: Perspect Public Health. 2023 Jun 29;145(1):25–31. doi: 10.1177/17579139231180746 (PMC11800687; doi:10.1177/17579139231180746)
Supplement: sj-docx-7-rsh-10.1177_17579139231180746 – Supplemental material for A systematic review of the effectiveness of community-based interventions aimed at improving health literacy of parents/carers of children [file sj-docx-7-rsh-10.1177_17579139231180746.docx]

| **PICOS** | **Inclusion** | **Exclusion** |
| --- | --- | --- |
| Population | parents of all races, ethnicities, cultural groups, and ages with child(ren) up to the age of 18 years | Any study measuring health literacy in children rather than parental health literacy will be excluded. |
| Intervention | Any intervention aimed at increasing parental health literacy. Which could include but is not limited to:   - Self-help tools - Confidence building activities - Knowledge sharing - Peer support - Teach back methods | Any study with a focus on interventions which the author does not affiliate with improving health literacy. |
| Comparison | Studies including any comparator/control will be included. Experimental studies will use a non-intervention group as a comparison. Studies that use a pre/post design will use baseline measures as control. |  |
| Outcome | Any study where health literacy is a measured outcome. Due to the wide variety of health literacy definitions and measures, any intervention that provides a health literacy definition and validated pre-post measure of health literacy will be considered. | Any study where health literacy is not a measured outcome. |
| Setting | All health literacy interventions in a community setting. This includes in the parent/caregiver’s home, primary care, and outpatient settings. The delivery mode of intervention could include virtual/online, remote (telephone/text), or face to face. | Any health literacy intervention delivered in a clinical (inpatient) setting. |

**Supplemental Table 1.** Summary of inclusion and exclusion criteria
