# Supplementary material for: A systematic review of the effectiveness of community-based interventions aimed at improving health literacy of parents/carers of children
Source: Perspect Public Health. 2023 Jun 29;145(1):25–31. doi: 10.1177/17579139231180746 (PMC11800687; doi:10.1177/17579139231180746)
Supplement: sj-docx-8-rsh-10.1177_17579139231180746 – Supplemental material for A systematic review of the effectiveness of community-based interventions aimed at improving health literacy of parents/carers of children [file sj-docx-8-rsh-10.1177_17579139231180746.docx]

**Supplemental Table 2.** Summary of included studies

| **1^st^ Author (Country, year)** | **Study design & purpose** | **Sample size & characteristics**  **(children’s characteristics)** | **Study setting** | **Intervention (underpinning theory)** | **Health Literacy measure (Validated/Amended)** | **Health Literacy results** | **Key Findings** |
| --- | --- | --- | --- | --- | --- | --- | --- |
| **Randomised Controlled Trials** | | | | | | | |
| Chu et al. (New Zealand, 2019) | RCT  To evaluate the effect of a text-messaging program (MyTeen) on promoting parental competence and mental health literacy for parents. | 221 parents  Intervention n=109  Female 96.3%  (52.3% male, all children aged 10-15 years)  Control n=112  Female 97.3%  (57.1% male, all children aged 10-15 years) | Text messages to parents’ mobile phone. | 1 daily, ≥ 160 charter text-message sent for 4 weeks. Text messages were adapted from the Parenting Strategies Program, an evidence-based parenting guide developed through (1) systematic review and meta-analysis of parental factors associated with adolescent depression/ anxiety and (2) a Delphi study of expert consensus on strategies parents can use to reduce their child’s risk of depression/ anxiety. | Mental Health Literacy Scale (only part of the tool used to assess knowledge of where to seek information from)  (Validated) | Baseline Mean (SD)  Intervention 15.9 (2.7)  Control 16.1 (2.7)  Post Intervention Mean (SD )1 month  Intervention 17.4 (2.0)  Control 16.6 (2.4)  3 months  Intervention 17.6 (1.9)  Control 16.7 (2.2) | Texting messaging was effective at increasing parental knowledge on where to seek support for teens. A universal approach minimised the stigma associated with accessing parenting support. |
| Lotto et al., (Brazil, 2020) | RCT  To evaluate the effectiveness of educational messages in the control of early childhood caries in low socioeconomic children. | 104 dyads of parents and children  Intervention n = 52  (46% male, Mean age 3.4 months)  Comparison  n = 52  (44% male, Mean age 3.6 months) | Text messages and audio narrations to parents’ mobile phone via WhatsApp Messenger. | 13 text messages and audio recordings sent over 6 months. One text message and audio recording sent every two weeks on the theme of oral health. (Health Belief Model) | The eHealth Literacy Scale (eHEALS)  (Validated) | Baseline Mean (SD)  Intervention 24.02 (8.14)  Control 24.53 (8.50)  Post Intervention Mean (SD)  Intervention 26.39 (10.10)  Control 23.73 (9.79) | Intervention significantly increased parental eHEALS scores. Parent-orientated WhatsApp messages can contribute to oral health education. |
| Güven et al.,  (Turkey, 2020) | RCT  1) Develop and test a web-based epilepsy education program (WEEP) for youth with epilepsy and their parents.  2) To evaluate the efficacy of the WEEP by assessing knowledge, seizure self-efficacy, attitude, and eHealth literacy of youth with epilepsy and their parents. | 70 parents  Intervention n = 35  69% female  Mean age 39.6  (57% male, mean age 13.1 years)  Control n = 35  86% female  Mean age 40.1  (54% male, mean age 13.4 years) | Pre and post assessments carried out in outpatient clinic. 12-week access to WEEP in child/parents’  home via personal computer | 12 weeks unlimited access to the web-based epilepsy education program (WEEP).  A weekly reminder sent via text message to encourage use of website. Text messages included information regarding technical support for website.  Technical support was provided every two weeks via phone. | The eHealth Literacy Scale (eHEALS)  (Validated) | Baseline Mean (SD)  Intervention 2.61 (0.97)  Control 2.89 (0.74)  Post Intervention Mean (SD)  Intervention 3.72 (0.49)  Control 2.91 (0.70) | Intervention significantly increased parental eHEALS scores. The WEEP was effective in improving knowledge, self-efficacy of youth with epilepsy and their parents. |
| Otsuka-Ono et al. (Japan, 2019) | RCT  Evaluate the effectiveness of an immunization education program designed to meet the needs of mothers in Japan | 171 women  Intervention n=87  Mean age (SD)  Mother 32.8 (3.9)  Control n=84  Mean age (SD)  Mother 33.0 (4.9)  (All children ~ one month) | Outpatient clinic in a private hospital. | Two brief private in person sessions with the researcher. Guidebooks on infant immunization schedules were provided at first contact. At the second contact parents were asked what actions they had taken in relation to immunization since first contact. | Adapted scale of health literacy with permission from the scale’s developer. | Baseline Mean (SD)  Intervention 3.3 (0.8)  Control 3.4 (0.6)  Post-intervention Mean (SD)  Intervention 3.7 (0.7)  Control 3.6 (0.6) | The intervention improved immunization health literacy and vaccination rates. |
| **Non-randomised studies with comparison group** | | | | | | | |
| Abdollahi et al. (Iran, 2017) | Quasi-experimental  To assess the effect of an educational program on promoting physical activity in postpartum women. | 80 postpartum women  Intervention n = 40  68% < 30 years  32% ≥ 30 years  Education less than college 23%  Control n = 40  75% < 30 years  25% ≥ 30 years  Education under college 20%  (All children < 3 months) | Community health care centre | Four 80-minute small group sessions.  3 theory based  1 practical – jogging  The intervention sessions were designed to cover four key skills areas relating to health literacy:  1)Spoken communication 2) Written communication  3) Self-management & empowerment  4) Support systems | Short Test of Functional Health Literacy in Adults  (Validated)  Rapid Estimate of Adult Literacy in Medicine (Validated) | Baseline Mean (SD)  Intervention 26.02 (6.11)  Control 24.92 (6.13)  Post Intervention Mean (SD)  Intervention 29.18 (5.19)  Control 25.55 (5.88)  Baseline Mean (SD)  Intervention 60.88 (2.78)  Control 60.20 (2.57)  Post Intervention Mean (SD)  Intervention 62.18 (2.48)  Control 60.60 (2.58) | Health literacy and physical activity significantly increased among the intervention group. |
| Azevedo et al. (Portugal, 2018) | Quasi-experimental  To assess the impact of a web-based gamification program on nutrition literacy of families and explore differences in impact by socioeconomic status | 189 families  Intervention n=106  Median age 36  Control n=83  Median age 37 | Web-based intervention in participants homes. | Web-based platform was developed as a social network environment. Weekly nutritional challenges were set. The platform contained videos, recipes, and an interactive space. Main topics:   - promotion of fruit and vegetable consumption - decrease sugar consumption - decrease salt consumption | Adapted Nutritional Literacy Assessment Instrument | Baseline Mean (SD)  Intervention 72.7 (16.2)  Control 66.4 (15.6)  Post Intervention Mean (SD)  Intervention 78.8 (15.6)  Control 67.8 (16.1) | Nutrition literacy was significantly higher than baseline following the intervention regardless of parental education and perceived income status. The gamified digital interactive process seems to be a useful educational tool. |
| Hurley *et al.,* (Australia, 2018) | Mixed methods  To develop, pilot, and evaluate a targeted parent mental health literacy intervention through community sports clubs. | 66 parents  Mean age 44.86±5.2 years  Intervention n=42  77% female  Control n=24  70% female  (All male adolescents) | Local sports club or university campus | One in person group workshop. Duration 55-80 minutes (mean=65 minutes). Activities included facilitator led discussions, how to guides, promotion of websites and supplementary materials. | Mental Health Literacy Scale  (Adapted) | Baseline Mean (SD)  Mental health literacy  Intervention 77.53 (9.28)  Control – not reported  Post Intervention Mean (SD)  Intervention 81.33 (6.67)  Control 80.53 (8.27) | Parents reported intervention content to be engaging and relevant to their needs.  There were no differences between groups on overall mental health literacy at 1 month follow-up. |
| Hurley *et al.,* (Australia, 2021) | Matched controlled trial  To evaluate the effectiveness of a mental health literacy intervention for parents delivered through community sports clubs. | 540 parents  Female n=321  Male n=219  Mean age=47.42±5.33 years  59.4% female  Intervention n=352  Control n=188  (Adolescents) | Most workshops took place at local sport club facilities while two workshops took place at other community venues and seven at a university campus. | In person group workshops. Group size ranged from 2 to 103 participants (average 20 parents). Facilitator led group discussions, videos, scenarios, guidelines on communicating with adolescents, pamphlets and signposting to resources. | Mental Health Literacy Scale  (Adapted) | Baseline Mean (SE)  Confidence & Knowledge  Intervention 8.06 (0.10)  Control 8.38 (0.13)  Stigmatizing attitudes  Intervention 18.71(0.32)  Control 19.10(0.41)  Attitudes that promote help-seeking  Intervention 38.77(0.43)  Control 38.60 (0.54)  Post Intervention Mean (SE)  Confidence & Knowledge  Intervention 8.63 (0.11)  Control 8.25 (0.19)  Stigmatizing attitudes  Intervention 19.54 (0.33)  Control 19.47 (0.53)  Attitudes that promote help-seeking  Intervention 38.98 (0.46)  Control 37.78 (0.73) | Results suggest that a brief mental health educational intervention can positively improve some components of parents’ mental health literacy. |
| **Non-randomised studies without comparison group** | | | | | | | |
| Brown *et al.,* (England, 2020) | Repeated measures evaluation  The purpose of the evaluation, was to assess whether the Parents and Communities Together-PACT intervention met its objectives  PACT objectives:  1)To use community-organized and -led methods to engage mothers from local populations  2)To improve maternal mental health and other health-related outcomes | 61 mothers  Highest Educational Qualification  Postgraduate degree 9.8%  Undergraduate degree 41%  BTEC/NVQ 16.4%  A level 11.5%  GCSE 9.8%  (all children aged 0-5 years) | The intervention was based in three local hubs: one church, one church-related center, and one community center. | 1) Weekly 2hr sessions “mumspace “for social support, which ran for the duration of the project (6 months). Parents participated in parent-led workshops, on topics including: immunizations, importance of play and returning to work.  2)Weekly 2hr session for 12 weeks “Parent University” coordinated by a health visitor and co-designed with parents, health visitors, and midwives, which was co-led by parents and professionals. | Newest Vital Sign UK (NVS-UK)  (Validated) | Baseline Mean (SD)  All participants 2.91 (2.00)  Low literacy sub-group 0.38 (0.51)  Intermediate sub-group 2.39 (0.50)  Adequate/high literacy sub-group 5.26 (0.87)  Post Intervention  Mean (SD)  All participants 3.02 (2.02)  Low literacy sub-group 1.54 (1.05)  Intermediate sub-group 2.48 (1.78)  Adequate/high literacy sub-group 4.68 (1.67) | A significant increase in HL was found for those with low HL scores at baseline compared to 6 months follow up.  Community-organised and community-led interventions in collaboration with statutory health services can improve maternal mental health and other health related outcomes. |
| Nye & Robinia (USA, 2019) | Mixed methods  To determine baseline oral health literacy levels for a sample of adult carers of young children living in rural communities and to determine the effectiveness of a community-based education program on increasing these baseline levels of knowledge. | 75 participants  Female n=40  Male n=17  Other n=3  Declined to answer n=4  Missing data n=11 (characteristics not reported for those with missing data) ­­ | The project took place in three rural communities in Michigan. Sessions were delivered during an oral health fair. | An hour-long presentation. The presentation was delivered by a pediatric dentist or a registered nurse. Nine topics were covered including: when to use a ‘sippy’ cup or bottle,  smoking in the home and when a child should first see a dentist/frequency of visits. | Revised Upper Peninsula Oral Health Literacy Assessment Survey | Baseline Mean  68.8  Post Intervention Mean  92.6 | There were statistically significant increases in oral health literacy following the implementation of the educational programme. |
| Jimenez et al. (USA, 2021) | Prospective cohort  To assess the feasibility and accountability of a telephone-based education and navigation program for Hispanic parents of children hospitalized with traumatic brain injury (TBI). | 14 Hispanic parents  86% female  Mean age 35  71% Education less than high school  (58% male  Mean age 9.7 years) | First stage of intervention in hospital prior to child’s discharge, follow-up telephone based | Brain Injury Education and Navigation (1^st^ BIEN)  One in-person session to introduce a booklet which contains 4 modules. Follow up phone calls from Patient Navigators.  (Social cognitive theory) | Short Form Health Literacy Scale  (Validated) | Baseline Mean (SD)  9.8 (3.8)  Adjusted, difference in mean at 3 months (95%CI)  -0.6 (0 to -1.3)  Adjusted, difference in mean at 6 months (95%CI)  0.9 (0.2 to 1.2)  Adjusted, difference in mean at 12 months (95%CI)  1.3 (0.6 to -2.0) | Gains in health literacy did not reach statistical significance. Findings support feasibility and acceptability of intervention to facilitate transition of care for children with TBI. |

Abbreviations: RCT, Randomised controlled trial; SD, Standard deviation; SE, Standard error
